# Oxidative Stress and Immune Responses During Hepatitis C Virus Infection in *Tupaia belangeri*

**DOI:** 10.1038/s41598-017-10329-7

**Published:** 2017-08-29

**Authors:** Mohammad Enamul Hoque Kayesh, Sayeh Ezzikouri, Takahiro Sanada, Haiying Chi, Yukiko Hayashi, Khadija Rebbani, Bouchra Kitab, Aya Matsuu, Noriaki Miyoshi, Tsunekazu Hishima, Michinori Kohara, Kyoko Tsukiyama-Kohara

**Affiliations:** 1 Department of Pathological and Preventive Veterinary Science, The United Graduate School of Veterinary Science, Yamaguchi University, Yamaguchi, Japan; 2 Laboratory of Animal Hygiene, Joint Faculty of Veterinary Medicine, Kagoshima University, Kagoshima, Japan; 3 Transboundary Animal Diseases Centre, Joint Faculty of Veterinary Medicine, Kagoshima University, Kagoshima, Japan; 4 Virology Unit, Viral Hepatitis Laboratory, Institut Pasteur du Maroc, Casablanca, Morocco; 5 Department of Microbiology and Cell Biology, Tokyo Metropolitan Institute of Medical Science, Tokyo, Japan; 6 Department of Pathology, Tokyo Metropolitan Komagome Hospital, Bunkyo-ku, Tokyo, Japan; 7 Department of Animal Pathology, Joint Faculty of Veterinary Medicine, Kagoshima University, Kagoshima, Japan

## Abstract

Hepatitis C virus (HCV) is a leading cause of chronic liver disease, cirrhosis, and hepatocellular carcinoma. To address the molecular basis of HCV pathogenesis using tupaias (*Tupaia belangeri*), we characterized host responses upon HCV infection. Adult tupaias were infected with HCV genotypes 1a, 1b, 2a, or 4a. Viral RNA, alanine aminotransferase, anti-HCV core and anti-nonstructural protein NS3 antibody titres, reactive oxygen species (ROS), and anti-3β-hydroxysterol-Δ24reductase (DHCR24) antibody levels were measured at 2-week intervals from 0 to 41 weeks postinfection. All HCV genotypes established infections and showed intermittent HCV propagation. Moreover, all tupaias produced anti-core and anti-NS3 antibodies. ROS levels in sera and livers were significantly increased, resulting in induction of DHCR24 antibody production. Similarly, lymphocytic infiltration, disturbance of hepatic cords, and initiation of fibrosis were observed in livers from HCV-infected tupaias. Intrahepatic levels of Toll-like receptors 3, 7, and 8 were significantly increased in all HCV-infected tupaias. However, interferon-β was only significantly upregulated in HCV1a- and HCV2a-infected tupaias, accompanied by downregulation of sodium taurocholate cotransporting polypeptide. Thus, our findings showed that humoral and innate immune responses to HCV infection, ROS induction, and subsequent increases in DHCR24 auto-antibody production occurred in our tupaia model, providing novel insights into understanding HCV pathogenesis.

## Introduction

Hepatitis C virus (HCV) is a major public health problem that infects approximately 130–170 million people worldwide^1, 2^. HCV causes chronic hepatitis and is a major cause of liver cirrhosis and hepatocellular carcinoma (HCC)^1^. The first line of immune defence against HCV relies on cell-intrinsic innate immunity within hepatocytes. The HCV genome is a single-stranded, positive-sense RNA genome. During viral replication, HCV is sensed as non-self by pattern recognition receptors (PRRs) in the host cell, which identify and bind to pathogen-associated molecular patterns (PAMPs) within viral products, leading to activation of innate and adaptive immune responses^3^. Both effective innate and adaptive immune responses are involved in the control of HCV infections^4^; however, the role of the humoral immune system in HCV clearance is still unclear^5^.

Toll-like receptors (TLRs), an important component of innate immunity, play crucial roles in sensing invaders and initiating innate immune responses, thereby limiting the spreading of infections and modulating adaptive immune responses^6^. Sodium taurocholate cotransporting polypeptide (NTCP), a bile acid transporter expressed at the hepatocyte basolateral membrane^7^, can act as a regulator of antiviral immunity in HCV infection^8^. HCV infection can induce reactive oxygen species (ROS) production^9, 10^ and oxidative stress can lead to the formation of 8-hydroxydeoxyguanosine (8-OHdG), an indicator of oxidative DNA damage^11^. 3β-Hydroxysterol-Δ24reductase (DHCR24), a cholesterol biosynthetic enzyme^12^, is an essential host factor that plays a significant role in HCV replication^13^. Moreover, anti-DHCR24 auto-antibody levels are increased during the progression of HCV infection^14^. Indeed, the detection rate of HCC by anti-DHCR24 antibodies is higher (70.6%) than that of the standard HCC markers, alpha-fetoprotein (54.8%) or protein induced by vitamin K absence or antagonist-II (42.5%)^14^.

Recently, HCV can be completely cured by newly approved drugs, direct-acting antivirals (DAAs)^15, 16^. However, persistent hepatic inflammation, cirrhosis, and HCC have been reported in patients following viral clearance^17^. To solve these issues and develop an efficient vaccine against HCV, animal models are essential. Lack of small animal models is a great obstacle in the field of HCV research. To date, chimpanzees have been used as infection models for HCV. However, high costs and ethical concerns have restricted the use of chimpanzees in experimental infections. Recently, humanized chimeric mice^18^ and genetically humanized mice^19^ have been developed for use in HCV infection models^13, 18^. However, the use of mice has some disadvantages, including high cost, immunocompromised animal status, donor-to-donor variability, and inability to examine chronic infections. *Tupaia belangeri* belongs to the *Tupaiidae* family, which contains four genera and 19 extant species^20^. The evolutional characterization of 7 S RNA-derived short interspersed elements (SINEs) has shown that tupaias possess specific, chimeric Tu type II SINEs and can be clustered with primates^21^. Thus, genomic analysis suggested that tupaias are more closely related to humans than to rodents^21, 22^. Tupaias have been reported to be susceptible to several hepatotropic viruses that also infect humans, including hepatitis B virus^23, 24^, HCV^25, 26^, and hepatitis E virus^27^, and can be developed as an immunocompetent animal infection model. However, the molecular basis of HCV pathogenesis has not been fully characterized in the tupaia model due to a lack of characterization tools (e.g., specific antibodies, quantitative polymerase chain reaction [qPCR] assays, and cDNAs).

Therefore, in this study, we evaluated the susceptibility of tupaias to several viral strains of HCV and characterized the effects of HCV infection on ROS generation and its association with anti-DHCR24 antibody levels. We also characterized humoral immune responses to viral proteins and established a qPCR assay to evaluate TLR, NTCP, and cytokine expression to characterize the innate immune response during HCV infection, which may provide significant insight into HCV pathogenesis.

## Results

### Alanine aminotransferase (ALT) levels and viral loads in HCV-infected tupaia sera

**Figure 1:**
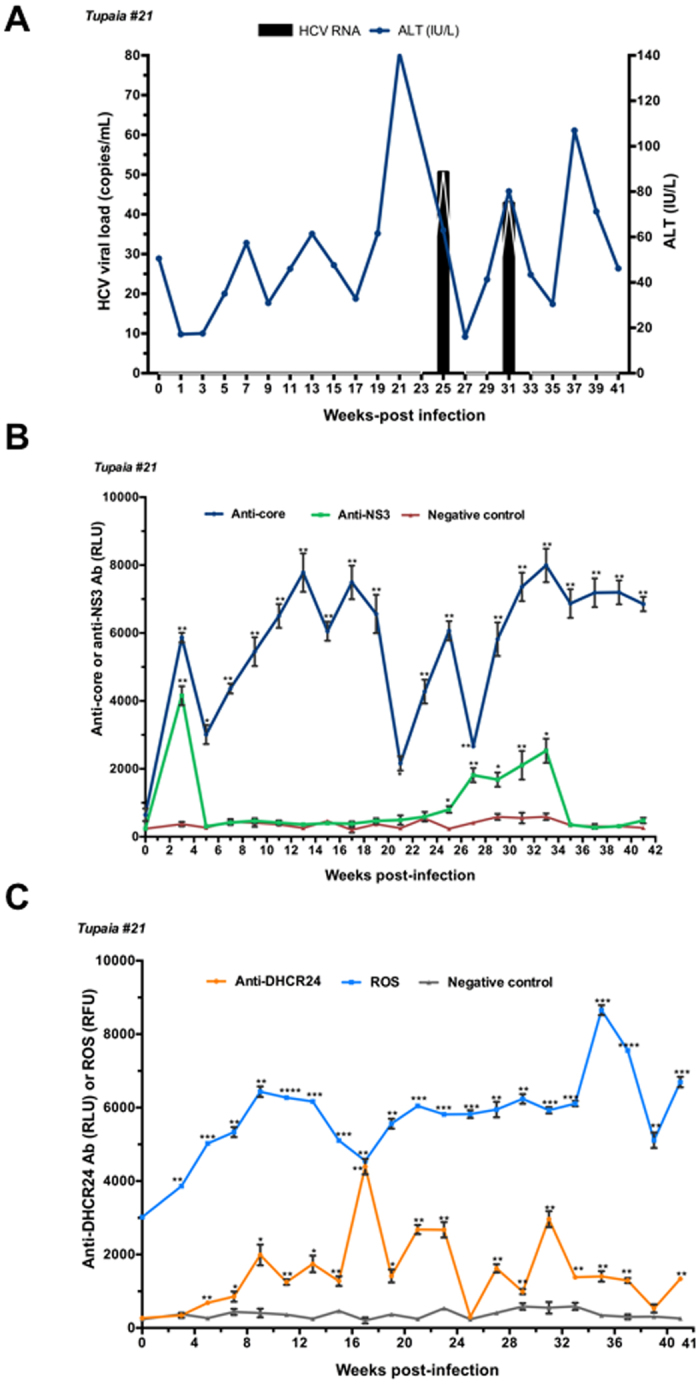
Response of tupaias to HCV1a infection. **(A**) ALT levels and viral loads in sera from tupaia #21 collected at 2-week intervals from 0 to 41 weeks postinfection (wpi). **(B**) Anti-HCV core and anti-nonstructural protein NS3 antibody titres in tupaia #21 at 2-week intervals from 0 to 41 wpi. **(C**) Anti-DHCR24 antibody titres and ROS levels in tupaia #21 at 2-week intervals from 0 to 41 wpi. The empty vector was used as the negative control. *p < 0.05, **p < 0.01, ***p < 0.001, and ****p < 0.0001 indicate significant differences in antibody titres or ROS levels, as appropriate, before infection and after infection at different weeks. Data are presented as means ± SDs (n = 2).

**Figure 2:**
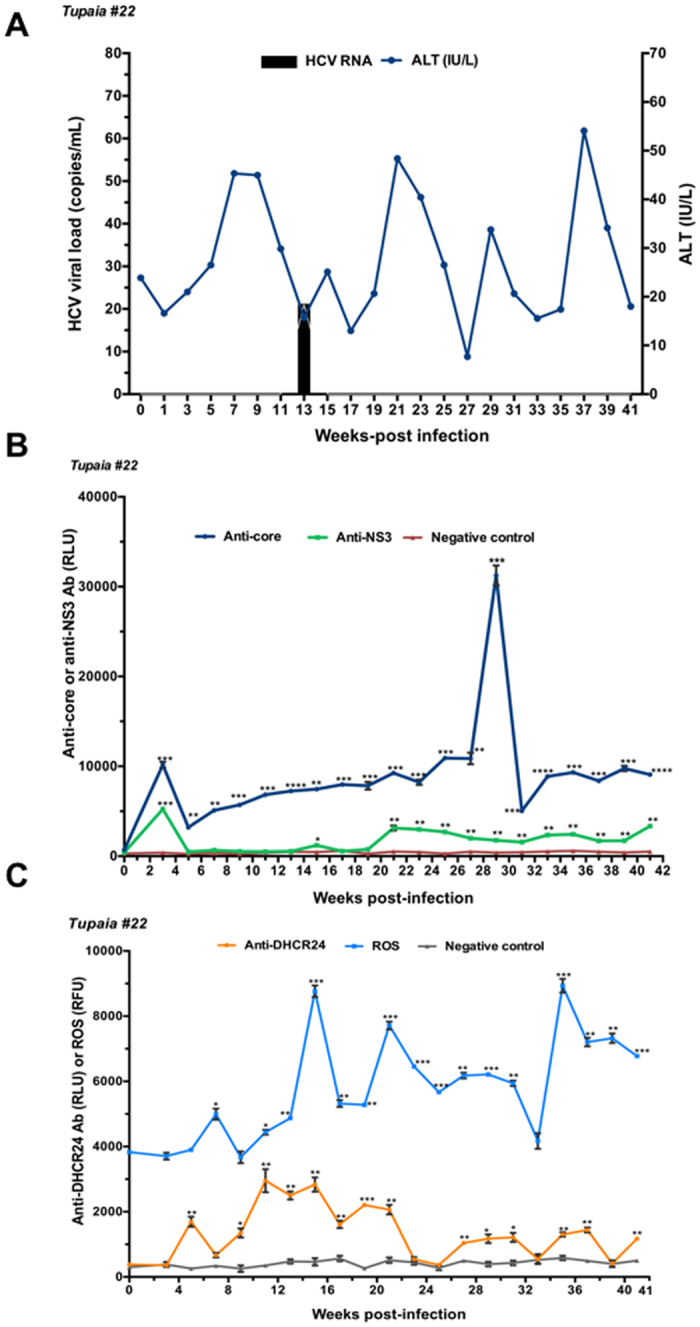
Response of tupaias to HCV1b infection. **(A)** ALT levels and viral loads in sera from tupaia #22 collected at 2-week intervals from 0 to 41 weeks postinfection (wpi). **(B**) Anti-HCV core and anti-nonstructural protein NS3 antibody titres in tupaia #22 at 2-week intervals from 0 to 41 wpi. **(C**) Anti-DHCR24 antibody titres and ROS levels in tupaia #22 at 2-week intervals from 0 to 41 wpi. The empty vector was used as the negative control. *p < 0.05, **p < 0.01, ***p < 0.001, and ****p < 0.0001 indicate significant differences in antibody titres or ROS levels, as appropriate, before infection and after infection at different weeks. Data are presented as means ± SDs (n = 2).

**Figure 3:**
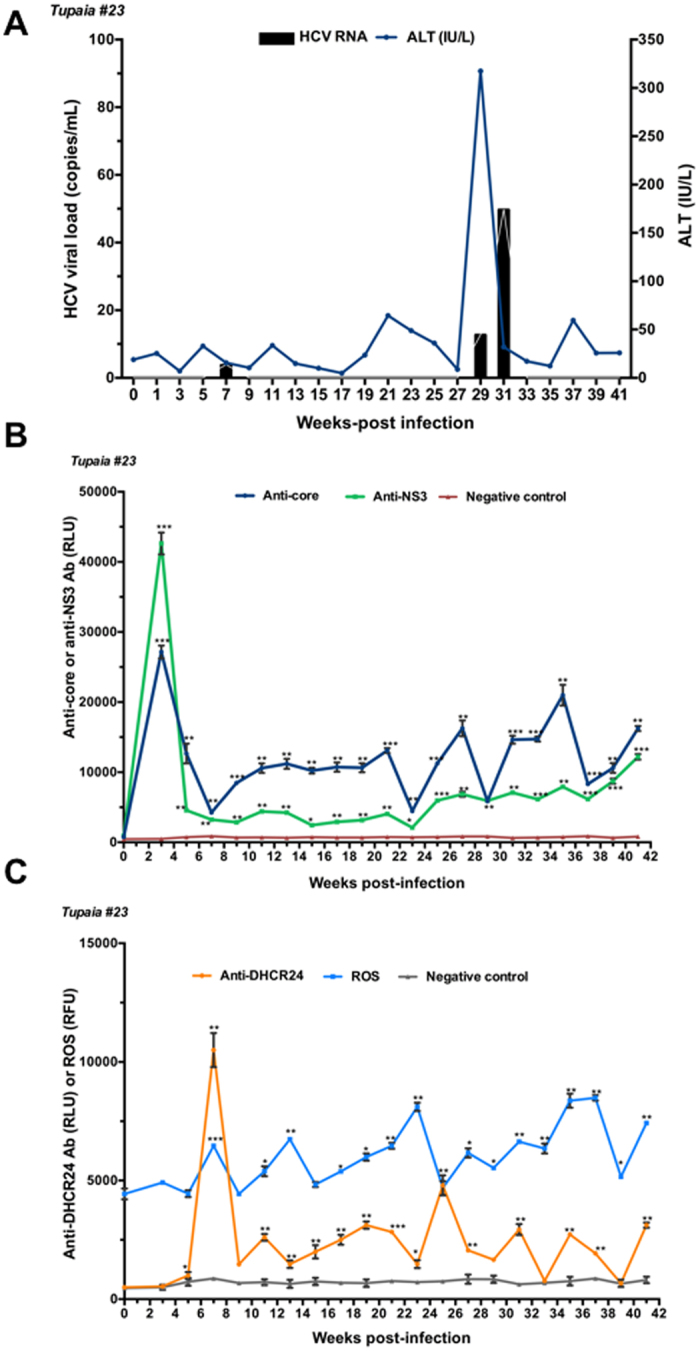
Response of tupaias to HCV4a infection. **(A**) ALT levels and viral loads in sera from tupaia #23 collected at 2-week intervals from 0 to 41 weeks postinfection (wpi). **(B**) Anti-HCV core and anti-nonstructural protein NS3 antibody titres in tupaia #23 at 2-week intervals from 0 to 41 wpi. **(C**) Anti-DHCR24 antibody titres and ROS levels in tupaia #23 at 2-week intervals from 0 to 41 wpi. The empty vector was used as the negative control. *p < 0.05, **p < 0.01, and ***p < 0.001 indicate significant differences in antibody titres or ROS levels, as appropriate, before infection and after infection at different weeks. Data are presented as means ± SDs (n = 2).

**Figure 4:**
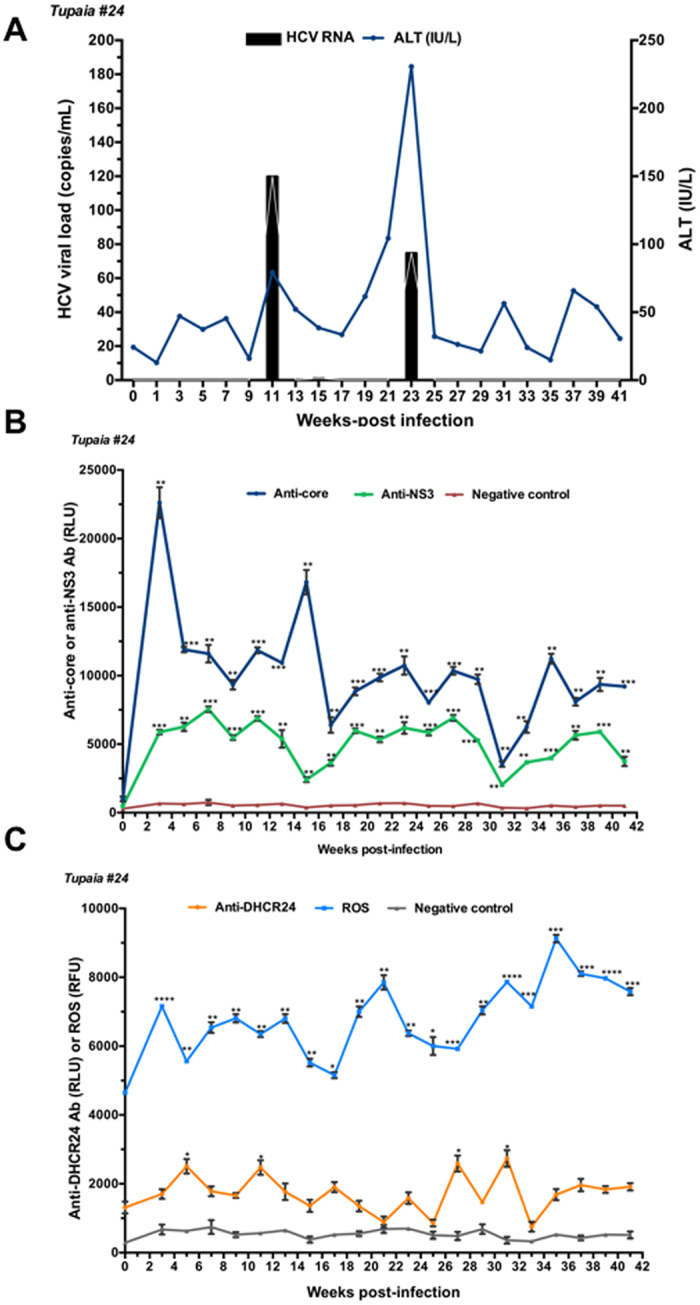
Response of tupaias to HCV2a infection. **(A**) ALT levels and viral loads in sera from tupaia #24 collected at 2-week intervals from 0 to 41 weeks postinfection (wpi). **(B)** Anti-HCV core and anti-nonstructural protein NS3 antibody titres in tupaia #24 at 2-week intervals from 0 to 41 wpi. **(C**) Anti-DHCR24 antibody titres and ROS levels in tupaia #24 at 2-week intervals from 0 to 41 wpi. The empty vector was used as the negative control. *p < 0.05, **p < 0.01, ***p < 0.001, and ****p < 0.0001 indicate significant differences in antibody titres or ROS levels, as appropriate, before infection and after infection at different weeks. Data are presented as means ± SDs (n = 2).

Tupaias were infected with HCV genotypes 1a (#21), 1b (#22), 4a (#23), and 2a (#24). The level of ALT fluctuated, and intermittent growth of HCV was observed in all tupaias (Figs 1A, 2A, 3A and 4A). The highest ALT level (317.5 IU/L) was observed in tupaia #23 at 29 weeks postinfection (wpi). Virus could be detected in serum at 25 (51 copies/mL) and 31 wpi (43 copies/mL) in tupaia #21; at 13 wpi (21 copies/mL) in tupaia #22; at 7 (4 copies/mL), 29 (13 copies/mL), and 31 wpi (50 copies/mL) in tupaia #23; and at 11 (120 copies/mL), 15 (2 copies/mL), and 23 wpi (75 copies/mL) in tupaia #24 (Figs 1A, 2A, 3A and 4A).

### Measurement of anti-core and anti-NS3 antibody titres

To investigate the course of HCV-specific humoral immune responses in HCV-infected tupaias, we measured the levels of anti-core and anti-NS3 antibody titres in serum samples before infection (0 wpi) and every 2 weeks from 3 wpi to until the tupaias were sacrificed at 41 wpi. Despite fluctuations, significant increases in anti-core antibody levels were observed in all tupaias from 3 to 41 wpi (Figs 1B, 2B, 3B and 4B). In tupaia #21, significant increases in anti-NS3 antibody titres were observed at 3 and 25–35 wpi (Fig. 1B). In tupaia #22, significant increases in anti-NS3 antibody titres were observed at 3, 15, and 21–41 wpi (Fig. 2B). Significant increases in anti-NS3 antibodies were observed in tupaias #23 and #24 from 3 to 41 wpi (Figs 3B and 4B).

At 3 wpi, though virus could not be detected in sera, a significant increase in anti-core and anti-NS3 antibody production was observed in all HCV-infected tupaias. Anti-core antibody production was decreased after the initial increase at 3 wpi, which further increased during or around peak viral propagation and fluctuated (Figs 1B, 2B, 3B and 4B). The highest anti-core antibody production was observed in tupaia #22 at 29 wpi (Fig. 2B). Additionally, anti-NS3 antibody production sharply decreased after 3 wpi and fluctuated. The highest anti-NS3 antibody production was observed in tupaia #23 at 3 wpi (Fig. 3B). Overall, peak NS3 antibody production was observed during or after peak viral propagation. Anti-core and anti-NS3 antibody titres in uninfected controls and positive controls were measured to ensure the specificity of the assay (Fig. S1A,B).

### Histological analysis of liver tissues from HCV-infected tupaias

**Figure 5:**
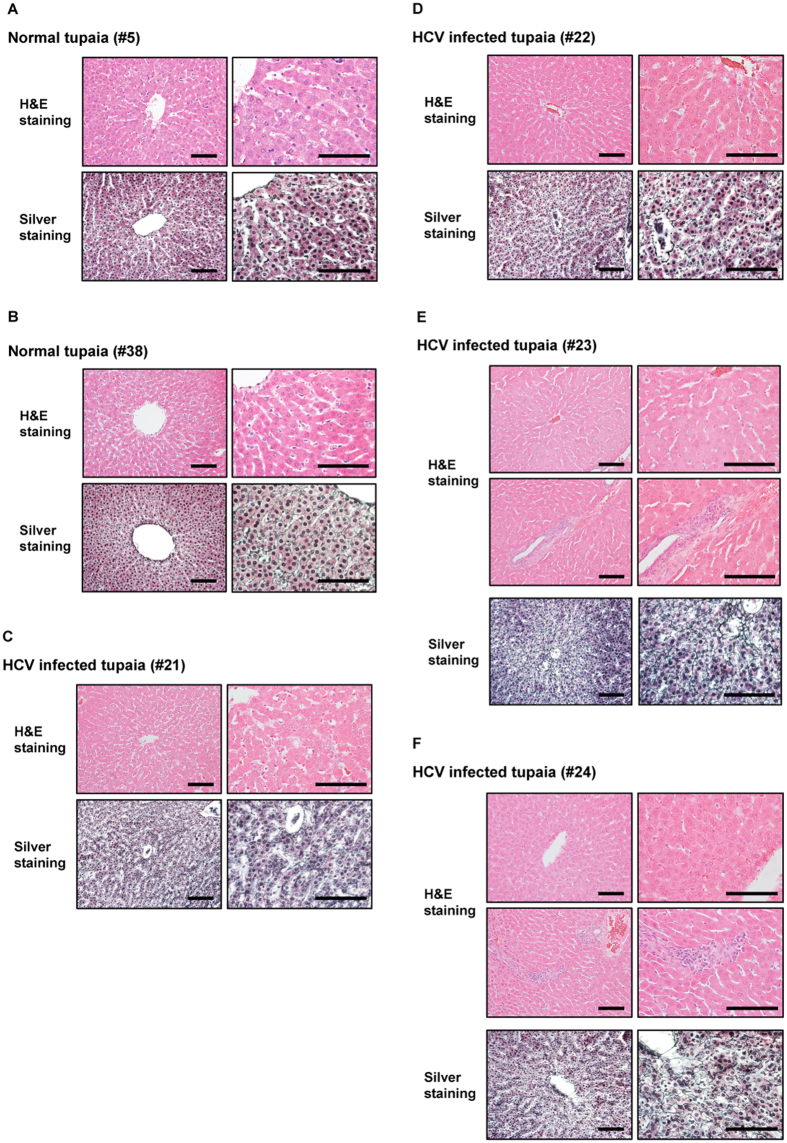
Histopathological analysis of liver tissues from normal and HCV-infected tupaias at 41 wpi. Histopathological analysis was performed with H&E or silver staining. H&E staining images (upper panel) and silver staining images (lower panel) of liver tissues from (**A**) Normal tupaia #5, **(B**) Normal tupaia #38, **(C)** HCV genotype 1a-infected tupaia #21, **(D**) HCV genotype 1b-infected tupaia #22, **(E)** HCV genotype 4a -infected tupaia #23, and **(F**) HCV genotype 2a -infected tupaia #24 have been shown. Black bars indicate 100 μm.

Histological analysis showed chronic hepatitis including abnormal architecture of liver cell cords, piecemeal necrosis, hepatocyte swelling, and lymphocytic infiltration into liver tissues of HCV-infected tupaias compared with that of normal tupaia liver tissues (Fig. 5). Silver staining showed increased fibres among hepatocytes (tupaias #21 and #22) (Fig. 5﻿C, D﻿) and thickened fibres (tupaias #23 and #24) (Fig. 5E, F), indicating the progression of fibrosis in HCV-infected tupaia livers.

### Measurement of ROS levels and anti-DHCR24 antibody titres

To investigate the effects of HCV infection on ROS generation, we measured ROS levels in sera from HCV-infected tupaias at 2-week intervals from 0 to 41 wpi. ROS levels were increased in all HCV-infected tupaias compared to ROS levels before infection (Figs 1C, 2C, 3C and 4C). ROS levels were also evaluated in mock-infected controls and with different concentrations of H_2_O_2_ as a positive control to ensure the appropriateness of the assay conditions (Fig. S1C).

To investigate whether increased ROS production induced anti-DHCR24 auto-antibody levels in HCV-infected tupaias, we measured the levels of serum anti-DHCR24 antibodies at 2-week intervals from 0 to 41 wpi. High levels of serum anti-DHCR24 antibodies were observed in all HCV-infected tupaias following ROS induction (Figs 1 C, 2 C, 3C and 4C). To ensure the specificity of the assay, anti-DHCR24 antibody titres in normal and positive controls were also evaluated (Fig. S1D).

### Measurement of oxidative stress in the liver

**Figure 6:**
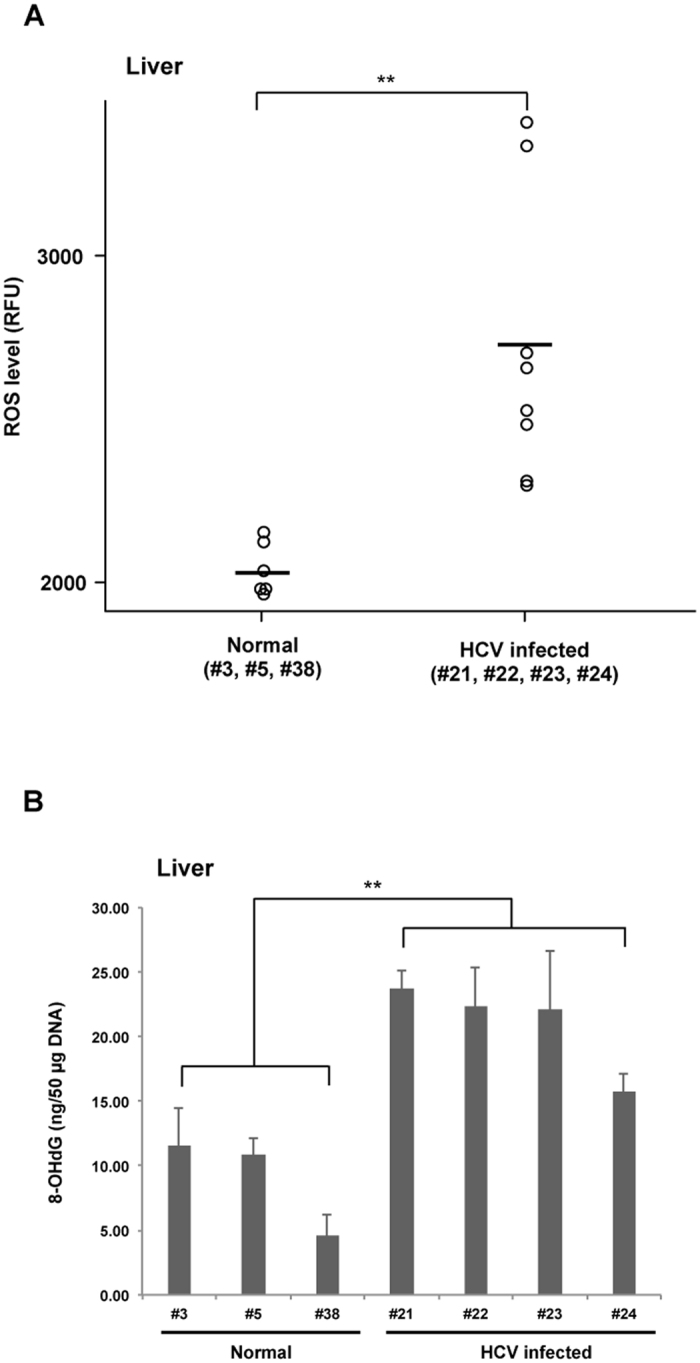
Intrahepatic ROS and 8-OHdG levels in normal and HCV-infected tupaias at 41 wpi. **(A)** ROS in liver lysates and (**B**) 8-OHdG levels in genomic DNA from HCV-infected tupaias were compared to those of normal controls. Horizontal bars in **(A)** indicate the mean value. **p < 0.01 indicates significant differences compared to normal controls.

To determine intrahepatic oxidative stress, we measured ROS levels in liver tissues at 41 wpi in normal and HCV-infected tupaias and found significantly higher ROS levels in HCV-infected tupaia liver tissues compared to normal controls (Fig. 6A). Additionally, at 41 wpi, we measured 8-OHdG levels in genomic DNA extracted from HCV-infected tupaia liver tissues to evaluate oxidative DNA damage. A significant increase in 8-OHdG levels was observed in all HCV-infected tupaias compared to normal controls (Fig. 6B).

### Changes in TLR, NTCP, and cytokine expression in liver tissues

**Figure 7:**
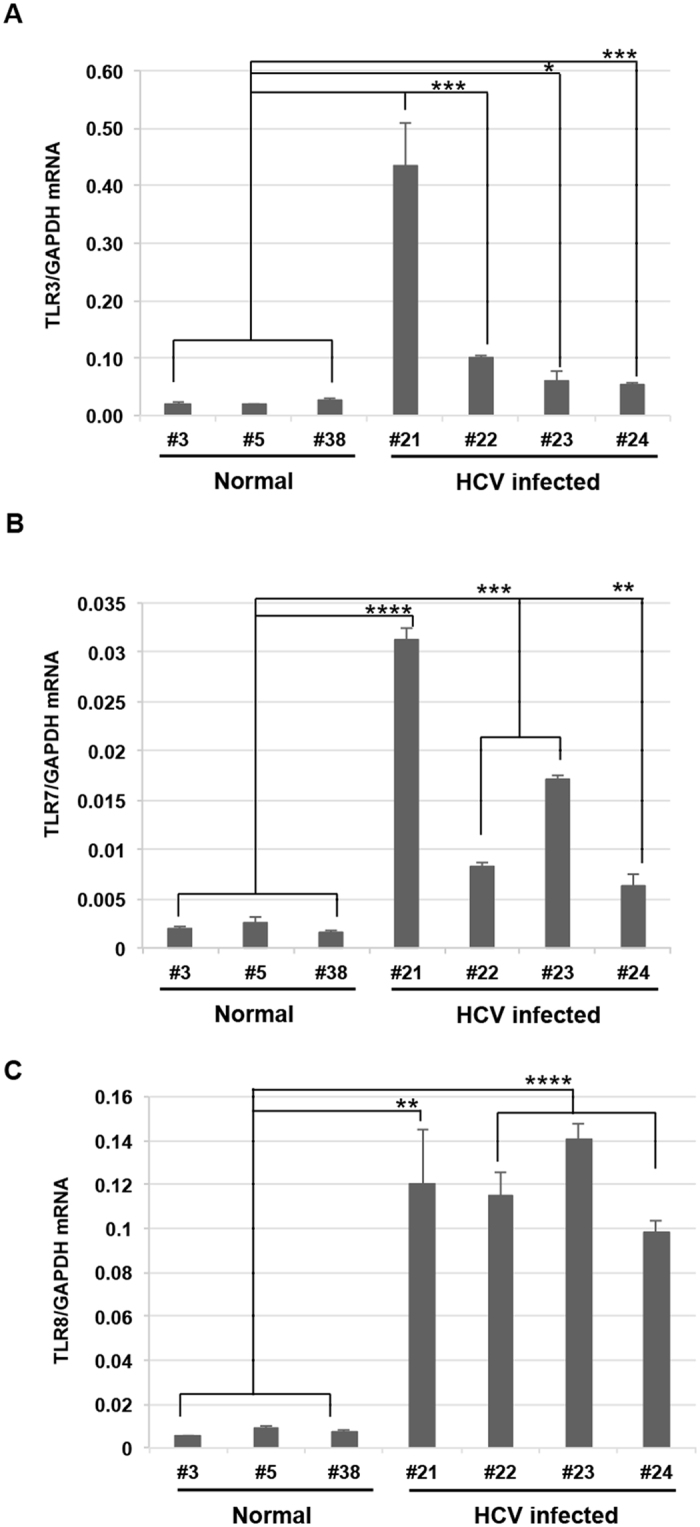
Changes in the expression of *TLR* mRNAs in HCV-infected tupaias at 41 wpi. (**A**) *TLR3*, (**B**) *TLR7*, and (**C**) *TLR8* mRNA expression in livers of HCV-infected tupaias was measured by one-step qRT-PCR. Gene expression levels were normalized to the expression level of *GAPDH* mRNA. *p < 0.05, **p < 0.01, ***p < 0.001, and ****p < 0.0001 indicate significant differences compared to normal controls. Data are presented as means ± SDs (n = 3).

**Figure 8:**
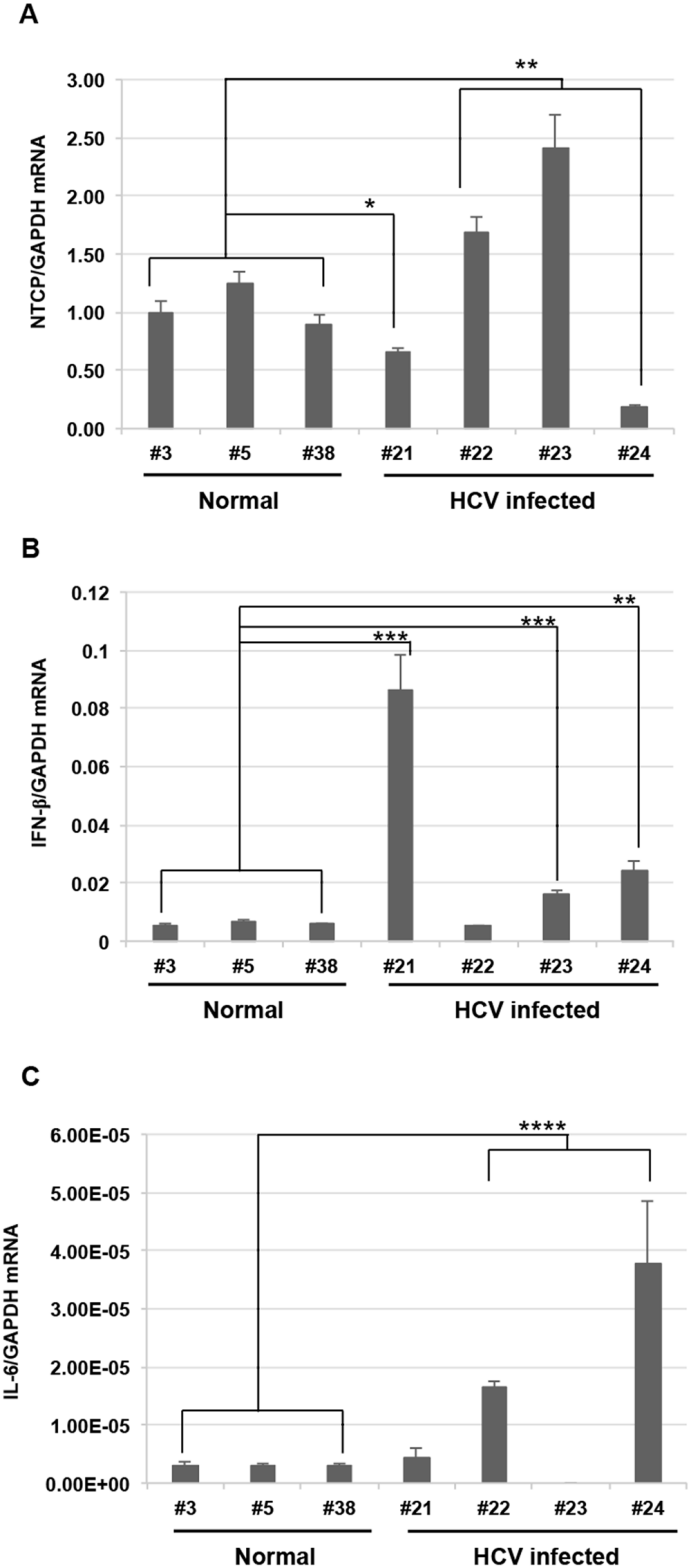
Changes in the expression of *NTCP* and cytokine mRNAs in HCV-infected tupaias at 41 wpi. **(A)** *NTCP*, **(B**) *IFN-β*, and **(C)** *IL-6* mRNA expression in livers of HCV-infected tupaias was measured by one-step qRT-PCR. Gene expression levels were normalized to the expression level of *GAPDH* mRNA. * *p* < 0.05, **p < 0.01, ***p < 0.001, and ****p < 0.0001 indicate significant differences compared to normal controls. Data are presented as means ± SDs (n = 3).

To investigate the mRNA expression patterns of TLRs, NTCP, and cytokines in liver tissues from HCV-infected tupaias, we isolated RNA from the livers of HCV-infected tupaias and measured the level of *TLR3*, *TLR7*, *TLR8*, *NTCP*, and cytokines (*interferon [IFN]-β* and *interleukin [IL]-6*) mRNAs at 41 wpi. Upregulation of *TLR3*, *TLR7*, and *TLR8* was observed in the liver tissues of all HCV-infected tupaias (#21, #22, #23, and #24) compared to uninfected normal tupaias (#3, #5, and #38; Fig. 7). *NTCP* was significantly suppressed in tupaias #21 and #24 and significantly upregulated in tupaias #22 and #23 (Fig. 8A). Moreover, significant upregulation of *IFN-β* was observed in all tupaias, except tupaia #22 (Fig. 8B). *IL-6* levels were significantly increased in tupaias #22 and #24 (Fig. 8C).

## Discussion

In this study, we demonstrated, for the first time, that ROS levels were higher in sera and liver tissues from HCV-infected tupaias than in that from uninfected tupaias. These data were consistent with previous findings of higher ROS levels in sera^28^ and liver tissues^29, 30^ of HCV-infected patients. We also observed increased 8-OHdG levels in livers from HCV-infected tupaias. These data were consistent with previous findings of higher 8-OHdG levels in livers from patients with chronic HCV infections^31, 32^. Previous studies have found that ROS induces the upregulation of DHCR24^10, 33^. Consistent with these results, we showed that anti-DHCR24 antibody production was increased with ROS generation during the course of HCV infection in tupaias. Thus, the tupaia HCV infection model supported the possibility that anti-DHCR24 antibodies could be a valuable marker to monitor HCV infection *in vivo* and should be consistent with previous evidence demonstrating that anti-DHCR24 auto-antibodies could be a useful biomarker for hepatitis C progression^14^.

In this study, we also characterized humoral and intrahepatic innate immune responses in tupaias infected with different HCV strains. Anti-NS3 and anti-core antibodies have been reported to be predominant in chronic HCV infections^34^. In fact, at 3 wpi, we found that all infected tupaias produced anti-core and anti-NS3 antibodies but were negative for serum HCV RNA. In our previous study, we detected HCV RNA only in the liver after 172 wpi^26^; therefore, HCV may replicate in the liver but not be released into the serum via an unknown mechanism. A longitudinal study in humans, with a median follow-up of 7 years, also reported cases in which core antibody was positive but HCV RNA was negative^35^. Additionally, the highest anti-core antibody levels in tupaia #22 were observed at 29 wpi, indicating robust viral replication occurred somewhere in the tupaia body. At 41 wpi, upon histological analysis of liver tissues of HCV-infected tupaias, lymphocytic infiltration was observed, indicating the occurrence of inflammation in the liver.

TLRs are important initiators of cytokine production, and the TLR signalling pathway serves as a link between innate and acquired immunity^36, 37^. TLRs act as PRRs for recognizing HCV PAMPs^38^. In our study, we found significant increases in *TLR3*, *TLR7*, and *TLR8* mRNA levels in liver tissues in chronically HCV-infected tupaias with different genotypes. Our results highlighted that HCV could trigger innate immune responses in livers of chronically infected tupaias. Thus, our data confirmed previous studies reporting that HCV could be recognized by TLR3, TLR7, and TLR8 in cell cultures^39, 40^.

HCV mainly infects hepatocytes, and two PRRs (retinoic acid-inducible genes I [RIG-I] and TLR3) recognize HCV RNA to trigger production of multiple cytokines, including type I IFN. HCV develops strategies to evade these immune responses through several mechanisms; for example, the cleavage or relocalisation of IFN-β promoter stimulator 1 by HCV NS3/4 A protease inhibits RIG-I signalling^41–43^. Furthermore, NS3/4 A disrupts the TLR3 pathway by degradation of TIR-domain-containing adapter-inducing IFN-β^43^. HCV core inhibits IFN signalling by interfering with the Janus kinase/signal transducer and activator of transcription pathway^44^, and HCV NS5A blocks 2′5′ oligoadenylate synthetase and induces IL-8^45, 46^. In livers from HCV-infected tupaias, TLR3 was induced in all four tupaias, whereas downstream IFN-β induction was observed in three tupaias (#21, #23, and #24).

Silencing of NTCP could inhibit HCV infection, whereas overexpression of NTCP could enhance HCV infection in cell culture^8^. NTCP can act as a regulator of antiviral immune responses in the liver. Moreover, NTCP is associated with the IFN response, and increased NTCP expression could suppress interferon-induced transmembrane protein (IFITM)-2 and IFITM-3 expression, and vice versa^8^. In our study, we found downregulation of NTCP but upregulation of IFN-β in tupaias #21 and #24. Significant upregulation of NTCP was observed in tupaias #22 and #23, and upregulation of IFN-β was only observed in tupaia #23. Thus, further studies are needed to explore the detailed mechanisms involved in NTCP-IFN interactions in HCV infection.

In conclusion, the tupaia infection model developed in this study was an effective approach to analyse the pathogenesis of HCV infection. ROS generation induced by HCV infection may be a trigger for the generation of anti-DHCR24 antibodies. Production of anti-core and anti-NS3 antibodies and intrahepatic innate immune responses upon HCV infection by alterations of TLR, NTCP, and cytokine expression highlights the potential applications of the tupaia infection model for the evaluation of HCV pathogenesis. Furthermore, tupaias could be a suitable small animal model for the evaluation of vaccines. The findings of this study provide novel insights into HCV pathogenesis and virus-host interactions.

## Materials and Methods

### Animals

The tupaias used in this study were obtained from the Laboratory Animal Center at the Kunming Institute of Zoology, Chinese Academy of Sciences (Kunming, China). This study was carried out following the Guidelines for Animal Experimentation of the Japanese Association for Laboratory Animal Science and the Guide for the Care and Use of Laboratory Animals of the National Institutes of Health. All experimental protocols were approved by the institutional review boards of the regional ethics committees of Kagoshima University (VM15051 and VM13044).

Animals were individually housed in cages and fed a daily regimen of eggs, fruit, water, and dry mouse food. The animals were humanely handled in accordance with the Institutional Animal Care and Use Committee for Laboratory Animals.

### Virus infection

All animals were found to be negative for HCV by quantitative real-time detection (RTD)-PCR before viral infection. Four adult tupaias (#21, #22, #23, and #24) were infected with HCV (genotypes 1a, 1b, 4a, and 2a [JFH1]), as described previously^26^, and three normal tupaias (#3, #5, and #38), which were not infected with HCV, were used as controls. Briefly, tupaias (#21, #22, #23, and #24) were infected at 12 months of age under anaesthesia induced by intramuscular injection of ketamine hydrochloride and atropine at 50 mg/kg body weight prior to virus inoculation and bleeding. Inocula derived from chimeric mice were introduced twice intraperitoneally at 1.5 × 10^8^ copies/mL for genotype 1a, 10^7^ copies/mL for genotypes 1b and 4a, and 1.2 × 10^8^ copies/mL for genotype 2a (JFH1) for tupaias #21, #22, #23, and #24, respectively.

The infected animals were bled (0.5 mL) biweekly, and sera were separated, aliquoted, and immediately used or stored at −80 °C for further analysis. At 41 wpi, infected and control animals were sacrificed, and liver tissues were extracted. RNA from liver tissues was isolated using the acid guanidinium-phenol-chloroform extraction method and purified with an RNeasy Mini Kit (Qiagen, Valencia, CA, USA). For histological analysis, liver tissues were characterized by haematoxylin and eosin (H&E) or silver staining, as described previously^47^.

### Measurement of ALT levels and viral load

Serum ALT level was determined using a Transnase Nissui kit (Nissui Pharmaceutical Co. Ltd., Tokyo, Japan); data were standardized and represented in IU/L. RNA was isolated from sera (50 µL) using SepaGene RV-R (Sanko Junyaku Co., Ltd., Tokyo, Japan). HCV RNA levels were quantified using qRT-PCR, as reported previously^48^.

### *Gaussia* luciferase immunoprecipitation system (GLIPS) assays

To evaluate antibody levels, GLIPS assays were performed as reported previously^49^, with some modifications, using SureBeads Protein G magnetic beads (Bio-Rad, Hercules, CA, USA). Briefly, the HCV core gene (1b, nucleotides 341–759), HCV NS3 gene (1b, nucleotides 3991–4753), and tupaia *DHCR24* gene (nucleotides 31–1599) were subcloned into the *Gaussia* luciferase vector (pGLIP vector)^49^ and expressed in HEK293 cells with Lipofectamine LTX and Plus Reagent (Invitrogen, Carlsbad, CA, USA) according to the manufacturer’s instructions. At 24–48 h after transfection, cells were lysed with *Renilla* Luciferase Assay Lysis Buffer (1×) and centrifuged at 20,380 × *g* for 5 min at 4 °C, and supernatants were collected and used immediately or stored at −80 °C. The luminescence of the crude extract was measured for 10 s using the *Renilla* luciferase assay system (Promega, Madison, WI, USA) on the GloMax-Multi + Detection System (Promega).

Immunoprecipitation assays were performed in duplicate in 96-well plates, and 100-fold diluted serum (1 µL equivalent) was used. To prepare beads, 25 μL of the Protein G magnetic bead suspension was added to each well and washed with 200 μL phosphate-buffered saline (PBS) + 0.1% Tween 20 (PBS-T) three times using a DynaMag-96 Side Skirted plate (Invitrogen). Supernatants were removed using an aspirator. Next, 100 μL of diluted serum from HCV-infected or mock-infected tupaias or known antibodies (used as a positive control) of different concentrations (10, 100, or 1000 ng) was added to the beads and incubated for 30 min at room temperature on a rotator. The beads were then washed three times with PBS-T, and lysates containing corresponding antigen or negative control antigens (empty vector) of 10^7^ light units were added to each well. After 1-h incubation at room temperature on a rotator, beads were washed with PBS-T at least three times. Finally, the beads were transferred with 50 μL of PBS into plates to be read, and 50 μL of *Renilla* substrate-buffer mixture (1×) was added to each well using an injector system during luminescence measurement, as stated above. The antibody titre was expressed as relative light units (RLU).

### Quantification of ROS levels

Total free radicals in serum or liver samples from HCV-infected and uninfected control tupaias were measured using an OxiSelect *In Vitro* ROS/RNS Assay Kit (Cell Biolabs, Inc., San Diego, CA, USA) according to the manufacturer’s protocol. Each reaction was performed in duplicate. Different concentrations of H_2_O_2_ were used in each reaction plate as positive controls to confirm the test conditions. Sera were diluted in PBS (1:200), and 50 μL of the diluted samples was collected into each well for the assay. For tissue lysates, 30 mg of liver tissues was homogenised using TissueLyser LT (Qiagen) in 1 mL PBS and centrifuged at 10,000 × *g* for 5 min. The supernatant was diluted in PBS (1:100), and 50 μL of the diluted samples was used in each well for the assay. The stabilized, highly reactive 2′,7′-dichlorodihydrofluorescin (DCFH) form was oxidized by ROS in the samples, and a catalyst was added to accelerate the reaction. The fluorescence of the oxidized DCFH to 2′,7′-dichlorofluorescein (DCF) was proportional to the concentration of ROS in the samples. Green fluorescence was monitored at 525 nm excitation/580–640 nm emission using the GloMax-Multi + Detection System (Promega). The ROS level was expressed as relative fluorescence units (RFU).

### Determination of 8-OHdG levels in genomic DNA from liver tissues

DNA was extracted from frozen liver tissues using the phenol-chloroform extraction method. The level of 8-OHdG in extracted DNA was determined using the OxiSelect Oxidative DNA Damage ELISA Kit (Cell Biolabs, Inc.) according to the manufacturer’s protocol with some modifications. Briefly, 100 µg DNA was digested with 6 units of nuclease P1 for 2 h at 37 °C in a final concentration of 200 nM sodium acetate (pH 5.5). Then, the DNA reaction mixture was subjected to further digestion with 2 units of alkaline phosphatase for 1 h at 37 °C in a final concentration of 1M Tris (pH 7.5). Finally, the reaction mixture was centrifuged for 5 min at 6,000 × *g* and the supernatant was used for 8-OHdG ELISA assay. Each reaction was performed in duplicate. The 8-OHdG content in unknown samples was determined by comparison with predetermined 8-OHdG standard curves.

### Gene expression analysis by qRT-PCR

Tupaia TLR and cytokine mRNA expression levels were measured by qRT-PCR, as described previously^50^. Primers used to detect tupaia *NTCP* gene [GenBank accession number: JQ608471] were as follows: forward primer (5′-TGTGGGCAAGAGCATCATGT-3′) and reverse primer (5′-CACTGTGCATTGAGGCGAAA-3′), and the reaction conditions were similar to that used for *TLR8* ^50^. Tupaia *GAPDH* was used as an endogenous control for normalization of the results.

### Statistical analysis

To analyse the statistical significance of antibody titres and ROS levels in animals before and after infection and to determine gene expression differences between uninfected controls and virus-infected individuals, unpaired t-tests were conducted using GraphPad software (http://graphpad.com/quickcalcs/ttest1/). Differences with *P* values of less than 0.05 were considered significant. All tests were two-sided.

## Electronic supplementary material

Supplementary Figure 1
