# Supplementary material for: Oxidative Stress and Immune Responses During Hepatitis C Virus Infection in Tupaia belangeri
Source: Sci Rep. 2017 Aug 29;7:9848. doi: 10.1038/s41598-017-10329-7 (PMC5575003; doi:10.1038/s41598-017-10329-7)
Supplement: Supplementary file 1 — Supplementary Figure 1 [file 41598_2017_10329_MOESM1_ESM.pdf]

Supplementary Information

Oxidative Stress and Immune Responses During Hepatitis C Virus Infection in *Tupaia belangeri*

Mohammad Enamul Hoque Kayesh[1,2#], Sayeh Ezzikouri[3,4#], Takahiro Sanada[5], Haiying Chi[2,3], Yukiko Hayashi[6], Khadija Rebbani[2,3], Bouchra Kitab[2,3], Aya Matsuu[2,3], Noriaki Miyoshi[7], Tsunekazu Hishima[6], Michinori Kohara[5*], and Kyoko Tsukiyama-Kohara[1,2,3*]

[1] Department of Pathological and Preventive Veterinary Science, The United Graduate School of Veterinary Science, Yamaguchi University, Yamaguchi, Japan

[2] Laboratory of Animal Hygiene, Joint Faculty of Veterinary Medicine, Kagoshima University, Kagoshima, Japan

[3] Transboundary Animal Diseases Centre, Joint Faculty of Veterinary Medicine, Kagoshima University, Kagoshima, Japan

[4] Virology Unit, Viral Hepatitis Laboratory, Institut Pasteur du Maroc, Casablanca, Morocco

[5] Department of Microbiology and Cell Biology, Tokyo Metropolitan Institute of

Medical Science, Japan

[6] Department of Pathology, Tokyo Metropolitan Komagome Hospital, Bunkyo-ku, Tokyo, Japan

[7] Department of Animal Pathology, Joint Faculty of Veterinary Medicine, Kagoshima University, Kagoshima, Japan

[#] These authors contributed equally to the study.

*Correspondence should be addressed to: Dr Kyoko Tsukiyama-Kohara, Transboundary Animal Diseases Center & Department of Animal Hygiene, Joint Faculty of Veterinary Medicine, Kagoshima University 1-21-24 Korimoto, Kagoshima-city 890-0065, Japan

Tel: +81-99-285-3589; Fax: +81-99-285-3589; E-mail: kohara@vet.kagoshima-u.ac.jp

Dr. Michinori Kohara

Department of Microbiology and Cell Biology, Tokyo Metropolitan Institute of Medical Science, Japan

Tel: +81-3-5316-3232; Fax: +81-3-5216-3137; E-mail: kohara-mc@igakuken.or.jp

Legends to Supplementary Figures

Figure S1. Antibody titre and ROS levels in negative (mock-infected) and positive controls.

(A) Anti-core antibody titres (indicated as RLU) in uninfected tupaias (#3, #5, #38) and for rabbit anti-core polyclonal IgG (10, 100, or 1000 ng; RR8[51]). Antibody titres were measured by GLIPS assays. The empty vector was used as the negative control. Data are presented as means ± SDs (n = 2). **(B)** Anti-NS3 antibody titres (indicated as RLU) in uninfected tupaias (#3, #5, #38) and positive control serum (200-, 400-, or 4000-fold dilution). Antibody titres were measured by GLIPS assays. The empty vector was used as the negative control. Data are presented as means ± SDs (n = 2). **(C)** ROS levels (indicated as RFU) in sera from uninfected tupaias (#3, #5, #38) and with different concentrations of H_2O_2 (0.313, 1.25, 5, 10, 20, and 40 μM). ROS levels were measured using an OxiSelect In Vitro ROS/RNS Assay Kit (Cell Biolabs, Inc.) following the manufacturer's protocol. Data are presented as means ± SDs (n = 2). **(D)** Anti-DHCR24 antibody titres (indicated as RLU) in uninfected tupaias (#3, #5, #38) and DHCR24 goat polyclonal IgG (10, 100, or 1000 ng; Santa Cruz Biotechnology). Antibody titres were

measured by GLIPS assays. The empty vector was used as the negative control. Data are presented as means ± SDs (n = 2).

References

51 Machida, K. *et al.* Inhibition of cytochrome c release in Fas-mediated signaling pathway in transgenic mice induced to express hepatitis C viral proteins. *The Journal of biological chemistry* **276**, 12140–12146, (2001).

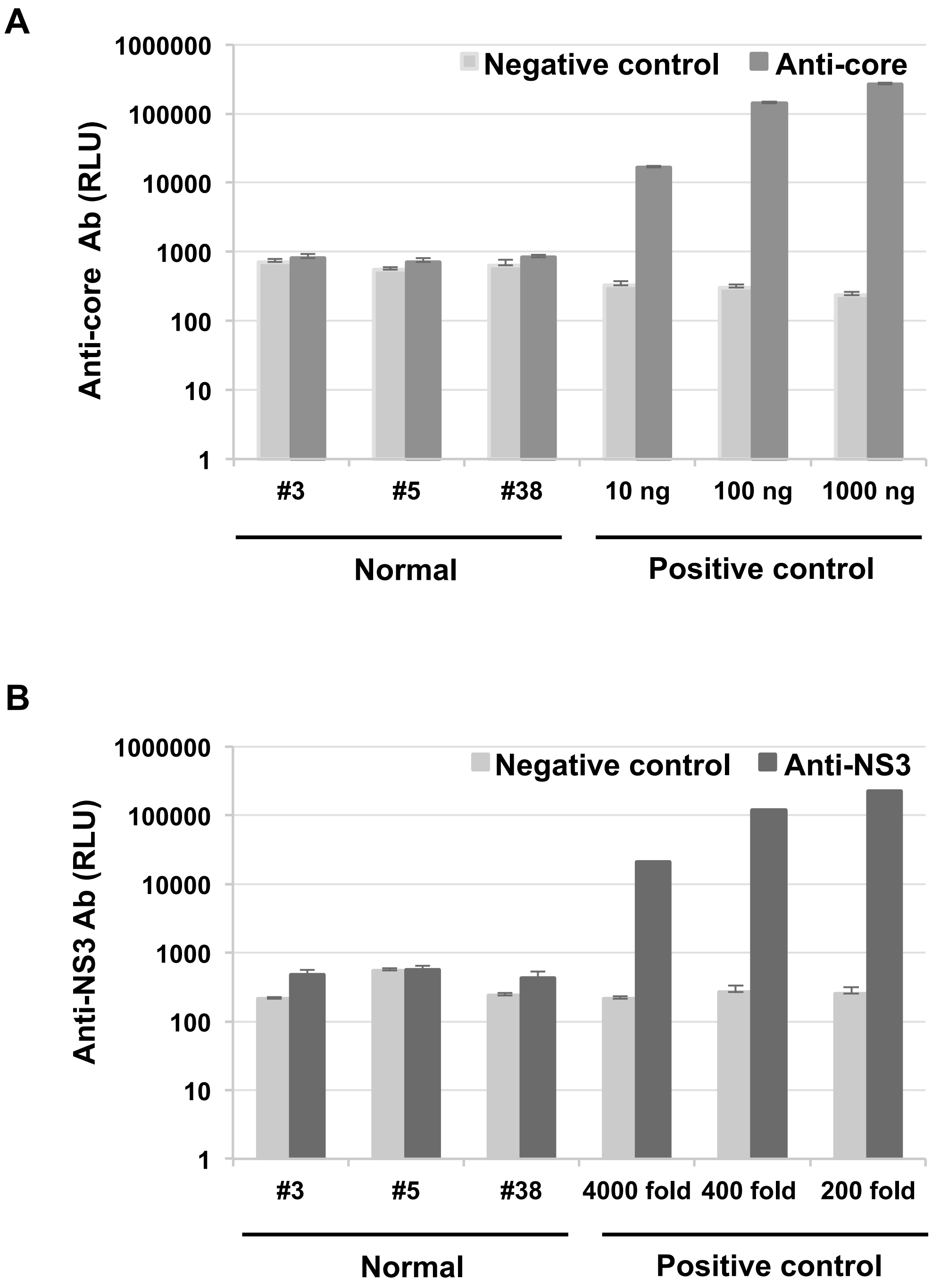
A
1000000
100000
10000
1000
100
10
1
Anti-core Ab (RLU)
Negative control
Anti-core
#3
#5
#38
10 ng
100 ng
1000 ng
Normal
Positive control
B
1000000
100000
10000
1000
100
10
1
Anti-NS3 Ab (RLU)
Negative control
Anti-NS3
#3
#5
#38
4000 fold
400 fold
200 fold
Normal
Positive control

Figure S1

C

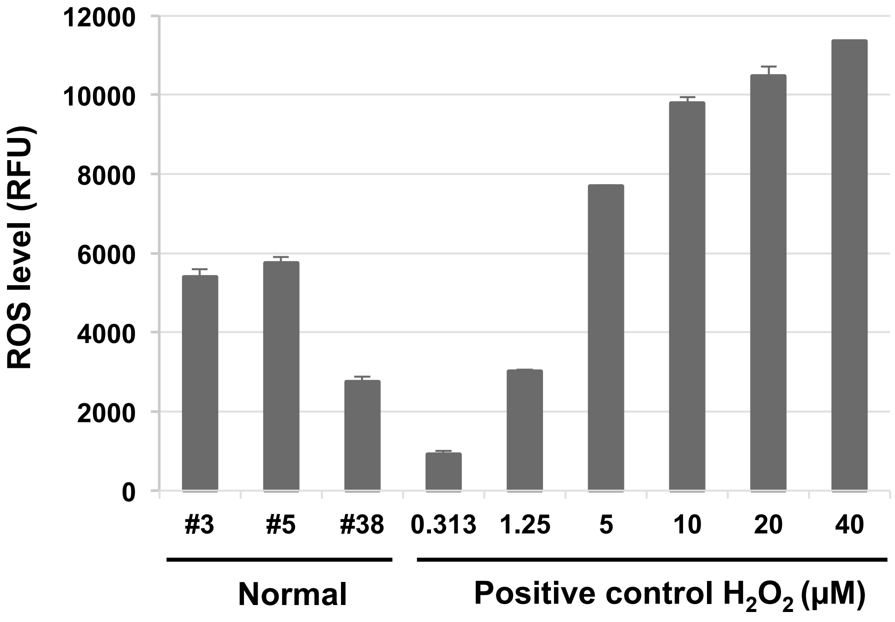
12000
10000
8000
6000
4000
2000
0
ROS level (RFU)
#3
#5
#38
0.313
1.25
5
10
20
40
Normal
Positive control H_2O_2 (μM)

D

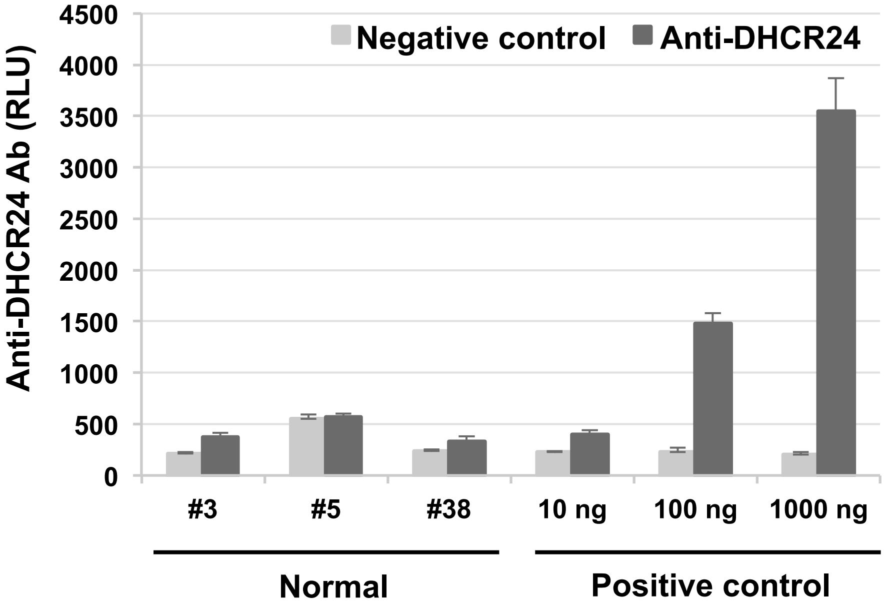
Negative control
Anti-DHCR24
4500
4000
3500
3000
2500
2000
1500
1000
500
0
Anti-DHCR24 Ab (RLU)
#3
#5
#38
10 ng
100 ng
1000 ng
Normal
Positive control

Figure S1
